# How Western Buddhist climate activists negotiate climate emotions

**DOI:** 10.3389/fpsyg.2024.1487258

**Published:** 2024-11-07

**Authors:** Johannes Cairns, Panu Pihkala

**Affiliations:** ^1^Faculty of Theology, University of Helsinki, Helsinki, Finland; ^2^Organismal and Evolutionary Biology Research Programme and Department of Computer Science, University of Helsinki, Helsinki, Finland; ^3^Department of Biology, University of Turku, Turku, Finland; ^4^Helsinki Institute of Sustainability Science (HELSUS), University of Helsinki, Helsinki, Finland

**Keywords:** religion and ecology, climate activism, Western Buddhism, climate emotions, ecological grief, eco-anger, eco-anxiety, eco-guilt

## Abstract

Understanding the underpinnings of pro-environmental behavior is key to mitigating the socio-ecological crisis. Climate emotions have a critical albeit complex role in modulating pro-environmental behavior. Moreover, ideological frames, particularly those from world religions, exert strong influence on pro-environmental behavior, covering most of humanity. Among these, Buddhism has long been argued to contain elements suited to a green transition. However, empirical research on Buddhism and ecology is scarce, and little is known about the dynamics between climate emotions and behavior among Buddhists. In this article, we increase knowledge about the complex dynamics of climate action by analyzing findings from a case study investigating thirteen Western Buddhist climate activists, who operate at the intersection of Buddhism and environmentalism. Life history and thematic interview data shows that interpretations of Buddhism shape attitudes toward climate emotions in profound ways, with respondents manifesting high levels of emotional reflexivity. Interpretations of compassion and interconnectedness facilitate various levels of care for non-human nature. Some participants reported climate anxiety. Teachings on impermanence and cultivation of equanimity affect engagement with climate grief, anger, despair, and hope. Interpretations on karma allow negotiating a balance between individual guilt and allocation of responsibility to social structures, although the role of climate guilt emerges as somewhat conflicted among participants. Furthermore, emotion norms on avoiding anger and conflict can prevent individual and collective activation, and some participants were critical about traditional Buddhist interpretations of anger. Withdrawal into Buddhist practice with an inner focus was used to cope with uncertainty and burnout, and when seen to address the psychological roots of the climate crisis this could facilitate social disengagement. Our study contributes to interdisciplinary research on climate emotions, environmental activism, and religion.

## Introduction

The current climate crisis shapes and interacts with human psychology and behavior in multiple ways. Understanding these links is also key to identifying circumstances inhibiting or conducive to pro-environmental behavior (PEB). For instance, compartmentalization of concerns, emotional detachment through hyperrationality, and emotional blocks to dealing with climate change can obstruct climate engagement even among those sufficiently informed (e.g., Gifford et al., 2018; Hoggett and Randall, 2018; Norgaard, 2011). Contrariwise, negative emotional responses to extreme weather have been identified as an important motivation for PEB (Ogunbode et al., 2018). Scholars have been increasingly interested in the various dynamics of eco-emotions and climate emotions, and their complex relationships with action, well-being, and functionality (e.g., Brosch and Sauter, 2023).^1^ In relation to environmental activists, it has been noted that ways of reacting to climate emotions have bearings on well-being, and lack of constructive coping with difficult emotions is one factor in activist burnout (e.g., Nairn, 2019). Various climate emotions felt by activists are a topic of growing research interest (e.g., Kleres and Wettergren, 2017; Martiskainen et al., 2020; Coppola and Pihkala, 2023).

Religions have been shown to significantly modulate human responses to the climate crisis (e.g., Haluza-DeLay, 2014). Religions affect environmental behavior directly through institutional practices and traditional as well as advocated lifestyles, which always have environmental implications, as well as indirectly through worldviews and values (e.g., Taylor, 2005). In the field of religion and ecology, increasing focus has been paid in recent decades on the impact of world religions^2^ on human environmental thought, behavior, and their interplay, since these have a major effect on the global potential of humanity for climate change mitigation.^3^ A seminal argument in the field was that proposed by historian Lynn White in *Science* in 1967, postulating that Abrahamic religions are the cause of anthropogenic environmental destruction due to placing human beings above non-human nature and nature as having only instrumental value as a resource for human beings to exploit (White, 1967). White proposed that by placing human beings as part of rather than above non-human nature, Eastern religions, notably Buddhism, could serve as a basis for more environmentally sustainable behavior. Advocating for the environmentally beneficial character of Buddhism and Eastern religions became a significant part of environmental discourses, which coincided with the rising interest toward these religions in the West and the common criticism of traditional institutions in the late 1960s and the 1970s (Nash, 1989).

However, scholars have observed that the relationship between Buddhism and environmental issues is complicated (Cairns, 2024), and empirical research on Buddhism and ecology is scarce. It is important to study religious doctrines not only through classical textual material and teachings by contemporary religious leaders but also through inspecting the behavior of adherents, since the doctrines highlighted by these dimensions can differ considerably (e.g., Jenkins, 2008). Empirical study on embodied religion can reveal, for instance, to what extent and which religious doctrines are reflected in the daily life of adherents, and how adherents navigate and negotiate tensions between religious doctrines and other socio-environmental demands (e.g., social norms, family demands, and economic constraints). In the context of religion and ecology, in particular, human thought has been shown to have a complex relationship with pro-environmental behavior (e.g., Haluza-DeLay, 2014). For instance, the environmental stances promoted by religious leaders may not reflect the environmental attitudes and behaviors of adherents, religion might play a negligible role or no role at all in motives for pro-environmental behavior among adherents, and there can also occur reverse causality whereby societal promotion of pro-environmental attitudes and behavior induces the development of religious ecophilosophies among adherents (Koehrsen and Huber, 2021). Furthermore, the dynamics between climate emotions and behavior among Buddhists have not been studied in depth.

In this article, we present findings from a case study investigating Western Buddhist climate activists operating at the intersection of Buddhism and environmentalism. We aim to increase understanding of the complex dynamics between religion, climate emotions, and environmental activism. Life history and thematic interview data from thirteen participants shows that interpretations of Buddhism shape attitudes toward climate emotions in profound ways, with respondents manifesting high levels of emotional reflexivity.

### Relevance of Western Buddhism for environmental psychology and behavior

Research in subsequent decades has both supported and challenged “the Lynn White thesis” (e.g., Whitney, 2013; Taylor, 2016; Taylor et al., 2016). The role of any single religion has been shown to be multifaceted. For instance, in addition to attitudes of domination, the social ethos of Christianity has led to important environmental action with increasing awareness of environmental issues, while the Buddhist tradition has emphasized rejecting the external world and its inevitable problems (Skt. *saṃsāra*) (Cairns, 2024; King, 2009). In traditional Buddhist contexts, this focus is seen in the laity as maintaining monastics to generate spiritual merit and in monastics as seeking enlightenment to escape *saṃsāra*. In the modern context and in the West, this focus is seen in Buddhists as seeking to enhance their personal peace of mind and wellbeing through meditation and study rather than engaging with the world at large, especially difficult issues which might obstruct one’s peace of mind (*cf.*, Loy, 2018). Buddhist doctrine has also been shown to be more anthropocentric than understood by White, perceiving human life as more precious than other sentient life and failing to recognize value in life beyond the observable suffering of individual sentient beings, such as the value of species, communities, and ecosystems (Schmithausen, 1997; Sciberras, 2008).

Similar to advocates of other religious traditions, since the environmental awakening of the 1960s, Buddhists have sought to reinterpret the tradition in response to anthropogenic environmental destruction. The development began at the intersection of emerging Western Buddhism and environmentalism, with many people in the West setting high expectations for the environment friendliness of Buddhism (e.g., Nash, 1989). Among the most prominent Buddhist ecophilosophers of this era, Joanna Macy redefined the Mahāyāna Buddhist practitioner ideal of a Bodhisattva, a being whose Buddhist practice is motivated by a wish to save all sentient beings from suffering, as an “Ecosattva” whose Buddhist practice is motivated by a wish to save ecosystems, maintaining all life, from destruction (Macy, 2009). This approach entails joining an innate affective sense of compassion with a tangible, internalized insight of the interconnectedness of all life forms, and their well-being, as part of ecosystems. It draws on various strands of Mahāyāna Buddhist teachings, with correspondences also in early Buddhist teachings. At the same time, Macy was one of the pioneers in developing awareness of eco-emotions and methods of engaging with them (e.g., Macy, 1983; Macy and Brown, 1998).

Western Buddhist ecophilosophies such as that by Macy have been influential (e.g., Stroebel, 2005) but also criticized as mixtures of eclectic US East Coast spirituality and selectively chosen and reinterpreted Buddhist elements (Harris, 1995). Nevertheless, several influential Buddhist figures, such as the Dalai Lama (Gyatso, 2020) and Thích Nhất Hạnh (Nhat Hanh, 2021), have also more recently called for Buddhists to respond to anthropogenic climate change through teachings and practices on compassion and interconnectedness. Stephanie Kaza, prominent scholar of Buddhism and ecology, has studied the potential for greening in Buddhism. Kaza argues that extending Buddhist elements on individual virtue ethics of compassion, nonharming, and skillfull means to encompass constructivist social ethics could be used in creative construction of responses for anthropogenic climate change both within and beyond Buddhism (Kaza, 2018). In this scheme, nonharming and compassion could be extended beyond individual virtues to cover social and environmental policy as well as structural problems related to sustainability. Skillful means, in turn, refers to the practice of assessing contextually appropriate action arising from nonharming and compassion. For the climate crisis, skillful means could entail different actions required to respond to effects of climate change at different levels (e.g., human society, animal conservation, ecosystem protection), also depending on the particular time and geographic location.

Recently, an increasing number of Western Buddhist actors have responded to the current global socio-ecological crisis. These include US philosopher and Zen teacher David Loy, who has criticized Buddhists for overt focus on personal otherworldly (e.g., enlightenment) or this-worldly (e.g., well-being, peace of mind) goals, described the climate crisis as a structural manifestation of individual dissatisfaction and craving, and called for a sustainability transition for Buddhism to be relevant for humanity in the future (Loy, 2018). Loy also founded the Rocky Mountain Ecodharma Retreat Center to develop sustainable Buddhism in practice (Gleig, 2021). Similar themes as those by Loy have been echoed in the teachings by renown Zen teacher Joan Halifax. She has a long history of social engagement on multiple issues and represents the US-originating, socially engaged Zen Peacemakers tradition which commonly features environmental activism (Gleig, 2021). Halifax describes the climate crisis as causing moral suffering in people due to the suffering in other beings associated with it, compromising the moral integrity of people, and she emphasizes the need for ‘wise hope’ as a way out of the moral suffering (Halifax, 2022). ‘Wise hope’ denotes an experience that what people can do matters, even if it only matters a little and it is uncertain how and when the fruits of the action will manifest. This is closely related to the concepts of ‘active hope’ coined by Joanna Macy (Macy and Johnstone, 2012) and ‘radical hope’ coined by Jonathan Lear (2006). Inspired by Macy’s concept, the US-based climate justice group One Earth Sangha founded by Insight Meditation practitioners Kristin Barker and Lou Leonard has developed “Ecosattva training,” as well as organizing a Dharma Teachers Statement on Climate Change (Gleig, 2021; Wamsler et al., 2018). Western Buddhist climate activist groups have also emerged as part of the New Climate Movement emerging especially since 2018, such as Extinction Rebellion (i.e., XR) Buddhists, which originated in the UK but has also spread to other Anglo-American countries (Gleig, 2021). However, virtually no field studies exist on this wave of emergent Buddhist environmentalism and climate activism, and little is known about how actors in these movements at the interface of Western climate activism and Western Buddhism combine these two aspects (see, however, Cairns et al., 2024).

What is particularly interesting in how study subjects combine Western Buddhism and climate activism are i. the tensions and negotiations involved, and ii. how combining the two aspects influences the practice of each, with special interest in those features facilitating or hindering the practice of each. Such findings have implications for both Buddhist practice and climate activism whose relevance might increase in the future in the case where either one or both of these increase in popularity in the West or across the globe. Here we focus on the role of climate emotions in these processes, namely, on how the experience, interpretation and management of climate emotions reciprocally modifies Western Buddhist practice and climate activism.

The question about how Buddhism influences and could influence PEB cannot be answered through philosophical analysis alone, especially taken the diversity of stances presented above and rapid recent developments in Western Buddhism. It also requires investigating the embodied ecophilosophies of Buddhists in the field. Of particular interest for this research are the embodied ecophilosophies of Buddhists operating at the forefront of environmentalism today, represented by New Climate Movement such as Extinction Rebellion (est. 2018) resulting from a heightened level of public climate awareness. In this article, we ask: How do Buddhists involved in such climate activism negotiate the conflicting environmental views and behaviors in their tradition? What are the implications of their Buddhist views and practices on their PEB, as well as the potential implications for PEB by other people within and beyond Buddhism? Our data naturally includes only a limited set of Buddhists, who may represent a marginal voice that has little bearing on Buddhist PEB at large, but it may also represent a pioneering voice working out the problem that others are bound to face soon, shedding light on the challenges and potential for greening in Buddhism and across humanity. This set of participants is also highly interesting in relation to the role of climate emotions.

### Climate emotions, pro-environmental behavior, and activism

Alongside and in interaction with ideology, the affective responses of people have a strong influence on environmental behavior. These can be, as described above, affective-cognitive responses from direct encountering of the devastating consequences of climate change, even causing clinical depression, anxiety, and post-traumatic stress disorder (PTSD). However, an increasing mass of evidence has accrued in recent years documenting a range of affective responses in humans in unaffected areas in conjunction with heightened public awareness of the climate crisis (e.g., Clayton and Manning, 2018). These responses have been studied under various fields of research, using various concepts, including affect, feeling, and emotion. Due to the lack of a unified scientific framework, all these have usually been broadly referred to as climate emotions: emotions which are significantly connected to anthropogenic climate change (Brosch and Sauter, 2023; Pihkala, 2022a).

Common climate emotions include grief, anxiety, fear, worry, despair, powerlessness, anger, guilt, care, connection, togetherness, and hope (e.g., Galway and Beery, 2022; Marczak et al., 2023; Pihkala, 2022a). Climate emotions manifest as these emotions do: for example, guilt has certain manifestations in human bodyminds regardless of the cause of guilt. However, the individual connotations, means of coping, and social context of climate emotions are so strongly linked to the features, effects, and mitigation of the anthropogenic climate crisis that it has become meaningful to discuss them as a separate cluster. The study of climate emotions is closely linked to the prior and more general study of environmental emotions, or eco-emotions, but again has the specific context of the climate crisis (e.g., Hamilton, 2020, 2022).

The affective responses of people to the climate crisis can have major implications for their environmental engagement, behavior, and well-being. However, these links are complex and partly context dependent. A certain climate emotion may manifest in various ways: for example, scholars have observed both constructive climate worry and paralyzing worry (for a review, see Ojala et al., 2021). Numerous social, psychological, and ecological factors can affect the dynamics of people’s climate emotions (see, e.g., the socio-ecological model by Crandon et al., 2022). For example, climate anger is fundamentally connected with action tendencies, but social circumstances may prevent a constructive expression of such moral outrage (e.g., Gregersen et al., 2023). It is also necessary to take various time frames into account. For example, intense climate grief may correlate with momentary withdrawal, but in the long run, it may be linked with formation of values and climate action (Ojala et al., 2021; Pihkala, 2024a). In general, many scholars, including us, are critical of the usage of the terms “positive emotion” and “negative emotion,” because these terms may be used in various connotations. Furthermore, sometimes “positive” emotions are argued to be more valuable than “negative” emotions. There can be many criteria for the evaluation of the valence of an emotion, and all kinds of emotions may have important functions (see Solomon and Stone, 2002; Wettergren, 2019; Bellocchi and Turner, 2019).

Climate emotions felt by activists have been a subject of a rapidly growing research interest (e.g., Martiskainen et al., 2020), and the complexity of climate emotions is gaining growing attention (e.g., Poma and Gravante, 2024). Scholars have been interested in many themes in relation to them: their connections with becoming an activist, the relationship between various climate emotions and various actions, and activist well-being (e.g., Pickard, 2021; Coppola and Pihkala, 2023). There has been an increased attention toward activist well-being and functionality, and climate emotions are closely related also to these aspects. If difficult emotions are not engaged with constructively, various problems may emerge: for example, grief may become complicated, helplessness can increase a paralyzing kind of anxiety, and guilt may become linked with shame and decrease efficacy (for discussions, see Hamilton, 2022; Pihkala, 2022a). Activism can be challenging in many ways, and there have been cases of burnout also in recent climate activism (e.g., Bird et al., 2024; Nairn, 2019). Investigating the ways in which climate activists engage with their emotions is important also in this regard. On a broad level, sociologists of emotion have studied emotion norms and societal dynamics of climate emotions (e.g., Neckel and Hasenfratz, 2021; Sauerborn, 2022), and for example youth scholars have warned against overly simplistic interpretations of climate emotions and activism (e.g., Bowman, 2019; Pickard, 2021). Sauerborn’s study is especially interesting in relation to Buddhism and our article, because it critically analyzes applications of mindfulness and emotion regulation by Extinction Rebellion.

Various terms have been used of ways to engage with emotions. Coping is a major framework here, and many studies have explored various ways of coping with climate change and coping with various climate emotions (e.g., Mah et al., 2020; Daeninck et al., 2023). The framework of emotion regulation has been less applied to this context, but it is receiving continuing interest generally. It can complement coping frameworks, for example, by bringing attention to ways in which people may select situations because of emotional motives, and to dynamics of either down-regulating or up-regulating emotional intensity (Gross, 2016; for application to environmental issues, see, e.g., Ejelöv et al., 2018; Kovacs et al., 2023). Abilities to engage with emotions constructively also have many names, including emotional literacy, emotional competence, and critical emotional awareness (e.g., Haase and Hudson, 2024; Ojala, 2022). People’s reactions to climate emotions may be evaluated to be either adaptive or maladaptive in relation to various standpoints. Sometimes a method of coping may be adaptive for individual well-being but maladaptive in relation to climate action and ethics. Research shows that the ability to name emotions and their nuanced forms, often called “emotional granularity” after Barrett (e.g., Barrett et al., 2001), is helpful for coping (Smidt and Suvak, 2015; Tan et al., 2022).

### Buddhism and climate emotions

The climate emotions people experience and the way they cope with them are influenced by many kinds of social, cultural, and psychological factors, including religion and spirituality. Furthermore, cultural and social emotion norms themselves may be shaped by ideology and worldviews. Both religious and secular ideological frameworks have emotional and behavioral norms affecting the way people express emotions and act upon them, conceptual frames affecting the meanings people give and the ways they relate to their emotions, and individual and collective practices affecting the intensity, duration, affective tone (positive, negative, or neutral), expression, and behavioral consequences of the emotions (which may also become transformed into other emotions) (e.g., Riis and Woodhead, 2010; for climate emotions and these kinds of factors, see, e.g., Crandon et al., 2022; Neckel and Hasenfratz, 2021).

These features are all relevant for the impacts of interpretations of Buddhism on (climate) emotions. In the Buddhist context, compassion and care (loving kindness) are considered particularly positive emotions, and equanimity and its expression is highly valued, as these features reflect the cultivation of ethical virtues and meditative states (e.g., Kaza, 2018). They particularly reflect lack of desire and ignorance, two of the three mental poisons in Buddhism, as ignorance means a failure to recognize the impermanent and non-self nature of conditioned phenomena, leading to self-centered grasping at objects and experiences. The same ignorance is also considered to result in anger, the third mental poison, originating from grasping at the desire not to encounter unpleasant or not to lose pleasant objects and experiences (e.g., Anderson, 2019; Cozort, 1995). Because of this difficult relationship with anger, Buddhist communities can have norms not to express anger or discuss and engage with difficult or confrontational topics that may give rise to anger. These may also include broader institutional cultures and structural forms of non-confrontation. These issues are likely to be strongly influenced by broader local societal norms, and could considerably differ between Buddhists in Western versus Asian societies or with corresponding cultural backgrounds. For instance, Anglo-American countries value assertiveness, while East Asian cultures value harmony and humility (Gelfand and Denison, 2020). These cultural differences combined with general features of the relation to anger and conflict within Buddhism are likely to result in diversity in the relation to anger between and within Western Buddhist communities, depending on the Buddhist tradition and its adherence to traditional Buddhist and Asian forms and customs. However, to our knowledge, little is known about this diversity in the field. Moreover, in the Buddhist context, grief can be considered to involve attachment to that which has been lost (Szántó, 2021), and guilt to be a form of anger directed at the self or to involve attachment to the self (Whitehead et al., 2018). These views are likely to influence the ways Buddhists relate to, interpret, and cope with climate emotions, as well as affecting the connection between the emotional states and PEB.

Buddhist individual and collective practices can also have a major impact on coping with emotions. Individual meditation and mindfulness practice has a mixed bag of results in terms of PEB (Adventure-Heart and Proeve, 2017: 111–113; Barbaro and Pickett, 2016; Barrett et al., 2016; Geiger et al., 2018, 2019). It can allow recognizing difficult climate emotions, moving beyond denial, as well as tolerating, accepting, calming, or transforming them, preventing or decreasing reactive and defensive psychological or behavioral impulses. Meditation and mindfulness practice may also exacerbate the experience of difficult emotions or bring unacknowledged negative mental states into the sphere of awareness, provoking a sense of panic, terror, despair, or depression. On the one hand, embracing negative climate emotions could make the individual connect personally with the climate emergency and motivate PEB. On the other hand, calming the disturbing emotions may lead to loss of motivating negative affect and thereby social passivity, and exacerbation of difficult climate emotions through meditation may lead to denial and avoidance behaviors. Furthermore, it is interesting to study whether mindfulness benefits recognition of climate emotions and whether the respondents have skills in emotional granularity.

Meditative practices around compassion, loving kindness, and interconnection may lead to connecting with the suffering caused by climate change and social engagement. Alternatively, they may function to primarily generate a positive emotional state of care and well-wishing in the mind of the practitioner in their sitting cushion without leading to physical engagement with the climate crisis potentially giving rise to less positive mental states. The effects of the same practices may be potentiated in a community context where people can share their experiences. Joanna Macy is a pioneer in developing Buddhist-inspired methods to deal with environmental emotions, and her methods have also been used in Buddhist communities, for instance, to embrace ecological grief and connect with non-human nature (Kaza, 2003, 2006; Kraft, 1994; Macy, 2009; Pihkala, 2020; Strain, 2016). However, these practices are marginally employed across Buddhist communities in the West and globally and are unlikely to reflect the climate emotion and behavioral implications of more conventional Buddhist practices. One typical collective ritual used across the Buddhist world is a chanting ceremony featuring sending positive energy and well-wishing to all sentient beings in the universe. In the conventional context, such ceremonies appear to have had little effect on environmental attitudes and behavior among Buddhists.

### Current study

In this study, we use a case study of thirteen Western Buddhist climate activists to investigate the influence of Buddhist elements on how they experience, express, and cope with climate emotions at individual and collective levels; their views and experiences from Buddhist communities; and the bearing these factors have on their pro-environmental behavior. We chose to focus on climate activists, who are likely to strongly engage with climate emotions, expecting material on climate emotions to be much richer compared to non-activists. We were also interested in how the study of Western Buddhists might reveal interesting dynamics of climate emotions, because of the Buddhist influence on encountering difficult eco-emotions (Joanna Macy, etc.) and the complex relationship about certain emotions (e.g., anger and guilt) in Buddhism. We wanted to contribute to several interdisciplinary research fields: climate emotions, environmental activism, and religion and ecology.

In-depth life history and thematic interview data for each participant was collected in 2022. The activists resided in Australia, Canada, the United Kingdom, and the United States and represented a wide variety of Buddhist traditions popular in contemporary Western societies. All of the participants were actively involved in Buddhist climate activist groups, with most having participated in Extinction Rebellion protests and several having been arrested during a protest. We analyzed the data for important experiences, climate emotions, coping mechanisms, emotion norms, and related Buddhist views and practices. In the discussion, we discuss the findings in relation to prior literature on these themes. We find that Buddhist views and practices have profound but manifold impacts on the expression, interpretation, coping with, and behavioral implications of climate emotions. We identify elements that could be used, in line with Kaza (2018), in the creative construction of responses for anthropogenic climate change both within and beyond Buddhism. Nonetheless, we also identify elements that can promote disengagement and disinterest, representing obstructions for the greening of Buddhism and its ability to advance greening at large.

We are interested in several topics related to climate emotions.

- What climate emotions do the participants report feeling significantly? How do they themselves see the relation between these emotions and their activism? What observations about these dynamics can scholars make on the basis of the data and interdisciplinary research literature?
- How does Buddhism influence the interpretations of climate emotions among these participants? What other factors influence their attitudes and reactions to climate emotions, and what is the interplay between (their view of) Buddhism and the other factors?
- Do the participants link Buddhism with a certain way of appraising certain climate emotions, and do they differentiate between various religions in this sense? Do they see certain strengths or weaknesses in Buddhist interpretations of climate emotions?
- Compared to the research literature on climate activism and emotions, what insights does this special group of people reveal?

## Materials and methods

The data collection procedure and demographics of the study participants have been described in detail previously (Cairns et al., 2024). Briefly, during 2021–2022, the lead author sought study participants involved in New Climate Movement activism (Buzogány and Scherhaufer, 2023; for definitions of activism and climate activism, see Corning and Myers, 2002; Kenis and Mathijs, 2012; Fisher, 2015) and self-defining as Buddhists through a combination of email and social media messaging, followed by snowball sampling.

The process led to the recruitment of 13 study participants from Canada, the United States, the United Kingdom, and Australia. The study participants all had university education, mostly lived in large cities, and were relatively evenly distributed in gender and age, ranging from their 30s to their 70s. The participants represented a large variety of Buddhist groups popular in the West, including Insight Meditation, Tibetan Buddhism, Zen, Pure Land Buddhism, secular Buddhism, and Triratna. Most participants had been practicing Buddhism for several decades, and only one participant had only very recently (under a year) become involved with Buddhist practice. Participating in Extinction Rebellion was the most common mode of climate activism, followed by the activities of Greenpeace and One Earth Sangha, a US based Buddhist climate engagement group. All participants were informed about the theme of the research project, processing and use of data, and their right to refuse to answer or withdraw at any point, expressing their consent either by signing a participant consent form or orally at the beginning of data collection.

The data was collected in 2022 through Zoom interviews carried out in English, performed once for each participant, and lasting approximately 2 h. The interviews were a combination of an unstructured and semi-structured part, including life history and thematic components. An initial unstructured part was used because of the lack of previous empirical research on the theme and the diversity of possible Buddhist and non-Buddhist motives for climate activism and experiences and interpretations of climate emotions, as well as the diversity of possible links between Buddhist and non-Buddhist elements (*cf.*, Cairns et al., 2024). An unstructured initial life story part was considered to allow for maximum freedom and richness in the individual framing of these issues, minimizing intentional or unintentional steering and potential biasing of the responses through the framing of the study questions by the researcher. A narrative (here, life story) framework can also facilitate self-reflection by the participant, producing more precise information compared to more structured interview approaches (Blackie et al., 2023). There has also been an increasing interest in studying narratives in eco-emotion research (e.g., Hickman, 2019).

The interviews started with, “Please could you freely describe your path into climate activism.” Depending on the contents and breadth of the initial answer, the lead author tailored further questions based on themes identified in literature. The main themes of interest pre-identified were: (1) the role of climate activism in the chronological life history and identity of the person; (2) the role of Buddhist elements in the climate activism; (3) biographical and structural availability of activism; and (4) political engagement of research participant. Theme 1 usually featured a question on the climate emotions experienced by the participant. Participants had been asked to reserve two hours for the interview. For one participant (P6: male, 70s, Australia), the allotted time ran out at the beginning of the semi-structured part and the participant had not addressed all the broad themes of interest in the life story part. Because of the methodology featuring a combination of unstructured and semi-structured parts, the particular additional questions asked from the participants in the latter part depended on the extent to which the broad themes of interest had been covered in the unstructured life story part. Therefore, each semi-structured interview was unique, with the specific questions and their wording varying between interviews, which is also a general feature of semi-structured interviews. This likely resulted in a trade-off favoring qualitative richness over detailed systematic coverage of each theme in the acquired data.

To protect the dignity of the research participants, it was considered important to ensure that no harm was caused to them during data gathering, storage, or reporting findings (Finnish National Board on Research Integrity TENK, 2023). Participation was based on informed consent, with information provided about the theme, aims, methods, and duration of the research as well as use and storage of the data. Participation was voluntary, and participants had the right to withdraw at any time before publishing the findings without any consequence. Because the interviews contain life history and event details, they cannot be anonymized for distribution in a public social science repository without removing a large fraction of the contents. Furthermore, they include highly sensitive data, such as information about criminal activity and arrests during climate protests. Therefore, to protect the anonymity of the research participants, the decision was made not to publish the data in a social science repository but only to share pseudonymized interviews with academic collaborators (see, e.g., Näre, 2022).

The lead author himself has experience as a Western practitioner of Buddhism, which helped to form trust with the participants, but naturally causes a need for critical reflection about how one’s positionality might influence the research setting and the analysis. Experiences in Western Buddhism may have influenced the choice of interview themes and the interpretation of research data in light of the vast scholarly literature on activism, environmentalism, Buddhism, and religion and ecology. For instance, the potential presence of negotiations regarding anger, compassion, and nonattachment/equanimity were expected based on personal experiences in diverse Western Buddhist contexts over several decades. The second author is not a Buddhist, and this outsider perspective was utilized in the analysis to ensure that the observations are as objective as possible. Both authors have specialized in religion and ecology studies, while the lead author has also specialized in study of Buddhist environmentalism and the second author on climate emotion research.

### Data analysis

To analyze negotiations by participants in relation to climate emotions, the lead author looked for important experiences and references to emotions. We use the term “emotion” to refer broadly to feelings, emotions, affects and moods, common to research on climate emotions, as discussed above in the Introduction. The lead author first used the taxonomy of climate emotions by Pihkala (2022a) to guide the coding of the data, looking particularly for mentions^4^ of grief, anxiety, anger, guilt, despair and hope, care/love, connection, and togetherness. These emotions were of special interest based on earlier research and our own chosen focus. Table 1 gives more information about the selection. This taxonomy influenced both data collection and data analysis, since during data collection, when asking about climate emotions, sometimes the lead author specified these to include a variety of emotions, enlisting, for instance, guilt, grief, anxiety, despair, hope, connection, and love.

**Table 1:** Chosen climate emotions and rationale behind the selection.

Emotion	Rationale
Grief and anxiety	Central climate emotions according to numerous studies (e.g., Ojala et al., 2021)
Anger	A climate emotion which has received growing research interest (e.g., Gregersen et al., 2023), and known to be conflicted in Buddhism
Guilt	A central climate emotion (e.g., Marczak et al., 2023)
Despair and hope	Subjects in many studies about climate emotions and motivation (e.g., Sangervo et al., 2022)
Care/love	Fundamental emotions related to motivation, and discussed in climate emotion research (e.g., Hickman et al., 2021)
Connection and togetherness	Important social emotions, which have so far received limited attention in climate emotion research (Pihkala, 2022a)

After the first author’s groundwork, the second author then went through the data and focused on the emotion-related content in his analysis. For each climate emotion, the lead author used a more inductive approach to subcode the Buddhist teachings and practices participants used to interpret and cope with the emotion, and the related negotiations concerning Buddhism and environmental activism. Finally, the authors used the way the participants described the climate emotions and their interrelations to critically examine previous literature on the theme. Thus, the coding process was refined iteratively in collaboration between both authors (see, e.g., Corbin and Strauss, 2008).

The interpretations of the results focus on the ways in which the participants negotiate climate emotions in relation, and especially on the role that Buddhist elements play in determining the relationship between climate emotions and pro-environmental behavior for the participants. We discuss the findings on this theme against a body of literature chosen based on the study interest of understanding how negotiation of climate emotions in the Buddhist context either facilitates or obstructs PEB. This body of literature includes previous work on Buddhism and ecology, climate emotions, motives for climate engagement, and studies on resilience and coping in relation to climate change.

## Results and discussion

The participants reported a wide array of different climate emotions. Some of them emerged very prominently, especially climate grief and compassion. Climate anger was notably debated by many participants, and there were interesting complications in relation to climate guilt. In the following, we analyze the participants’ views of selected climate emotions, including both difficult emotions such as anxiety and uplifting emotions such as togetherness.

### Anxiety

Climate anxiety has received growing attention since the late 2010s, together with climate grief (Cunsolo et al., 2020). As a broad term, it has been used to refer to an intertwined mix of various difficult climate emotions and mental states, such as distress, low moods, and fears (Pihkala, 2020). It can be differentiated from broader eco-anxiety, which can be caused by matters unrelated to climate change, but most people use these concepts interchangeably. Some scholars have focused on more specific characters of anxiety-as-emotion, pointing out that fundamentally eco-anxiety is related to perception of threats which include uncertainty (Kurth and Pihkala, 2022). People have different reactions to the term climate anxiety (Gregersen et al., 2024), and its usage depends on the cultural context (Sangervo et al., 2022). Some scholars prefer to speak about climate distress (Pihkala, 2024b).

Considering the prevalence of discussion on eco−/climate anxiety in media and research literature in recent years prior to the interviews, relatively few participants mention personal experiences with climate anxiety (four participants) or even awareness of the public discussion around it (one additional participant). Climate anxiety has been reported to be more prevalent among the youth compared to older generations (e.g., Hickman et al., 2021), and the scarcity of mentions may reflect the relatively advanced age of the participants in this study, with the youngest being in their mid-30s and several participants being in their 60s or 70s. In line with this, one participant refers to climate anxiety as something particularly affecting the youth.

*One of the things that I’ve been doing recently […] is to ask young people what they want from us, what they want us to do about climate emergency. The two responses I got was, stop debating, get on with the change […], and the second was, get rid of the politicians and start again. […] We need to listen to the young people about what they want, because they have lived with climate emergency from their first day. […] So, yes, there’s an anxiety (P12).*

Potentially reflecting the low attention paid to climate anxiety in general by the participants, there is also very little discussion about climate anxiety relative to Buddhist views and practice. However, the cultivation of equanimity and compassion through meditative practices was mentioned by many, also in the context of managing difficult climate emotions, and that may be considered to be useful for managing anxiety as well. Seeking to manage anxiety through meditation and mindfulness practice is typical in Western Buddhist and secular contexts and one of the prime targets of the most common form of therapeutic mindfulness practice (mindfulness-based stress reduction, MBSR) (Goldin and Gross, 2010). Taken this context into account, it is intriguing that eco/climate anxiety is mostly discussed in ways common in the Western context overall, which may also reflect that the more commonplace understanding and use of the term is a fairly recent phenomenon and its integration into Buddhist views and practice is therefore still at a very early stage. For instance, environmental grief, in contrast, has been discussed already for several decades, with Buddhist views and practices for addressing grief, formulated especially by Joanna Macy, having been around for equally long. The following passage illustrates an understanding of climate anxiety that is mostly based on a Western societal context without strong ties to Buddhism:

*I was feeling eco-anxiety for some years now before COVID, probably since 2018. That’s about the part where it started coming. Greta Thunberg was a major factor, bringing it to the international consciousness that you cannot just sit here and watch the Earth burn. We got to do something. As well as Dr. Jane Goodall, I think she was one of the first to say something. So eco-anxiety, that’s something I have and something a lot of people have nowadays. […] With eco-anxiety, it’s almost like a low-grade fever that burns you and kills you quietly and slowly, and will not go away. […] You’re always facing eco-anxiety and you do not see the powers be doing much about it, or they claim they are doing this and that, but they are not hitting any targets and you are always lying. That kind of eco-anxiety is pretty hard to handle, whether you are a Buddhist or something. I do not know if there’s any Buddhist training that is helping me handle that part, not directly (P7).*

Several participants engage in highly interesting discussions about the relationship between climate anxiety and other climate emotions. These discussions reflect partly different interpretations of what climate anxiety as a term means. One participant differentiates climate grief from climate anxiety and says that what they feel is mostly grief:

*It’s grief, grief is the one. I do not feel the anxiety as much […], I do not feel personally reasons for that. Maybe because it’s a fairly privileged middle-class existence, not living so far in a super affected, it’s not like I cannot imagine. But for me, it’s grief and it’s sadness (P9).*

Another participant emphasizes the interconnections between anxiety, grief, and guilt:

*Grief, anxiety and guilt. I’ve had them all, well, yes, yes, yes and yes. I do not know how to start. I’ll set aside guilt. So there’s grief and anxiety, which are almost clips of each other. I think as soon as you start taking climate issues seriously, unless you are braindead, you are automatically anxious. If you can connect your head and your gut, the anxiety arises immediately [when] you see what’s happening (P11).*

This participant highlights the role of anxiety as threat perception (in line with Kurth and Pihkala, 2022) and testifies to the close connections between these three broad emotions, which has come up also in other studies (e.g., Jensen, 2019; Pihkala, 2020). In a similar vein, another participant, who mostly relates experiences of climate grief, also describes a sense of overwhelm in the context of threat perception.

*It’s very easy at the moment to feel overwhelmed by just the enormity of the environmental damage and what’s happening. We’re about to get massive storms here in Victoria, which is really unusual for this time of the year (P13).*

One participant (they/them) mentions climate anxiety in connection with anxiety, depression, grief, and frustration both in general and in connection with the ecocrisis. They consider Buddhist practice, psychotherapy, and climate activism all to have helped in coping with these emotions. We include the following lengthy quote because it illustrates so well the interconnections between various topics.

*As a child and young adult, I was so emotionally locked down that whilst I was aware of the crisis intellectually, I did not feel a great deal of much in my own life. Or when I did feel things, depression, anxiety, things like that, frustration, whatever it was, it was all very muddled up and I would not have been able to separate what was my own personal process and psychodynamic process from my life history and from the different immediate situations that I was landing in and how I was reacting to those, to what I felt about world events, or the climate crisis or the ecological emergency. […] As my Buddhist practice and therapeutic practice progressed, it became easier to identify the different aspects of how I was reacting to that. […] The Buddhist practice beginning to address those deep insecurities and the personal therapy and the therapy training beginning to address those insecurities. [I would] let go more and more of the self-protection, of depression and anxiety, and low self-esteem. As those things are released and relaxed it’s more possible to act in the world in a meaningful way. Of course acting in the world in a meaningful way supports my mental health to be to be more balanced and to be better so there’s a kind of positive cycle I guess. […] I would say until I got involved with XR, there was a bit of a dance really that sometimes I would feel despair and then actually because I did not know what to do with that, I had to change it. I might occasionally work with it in a spiritual way or a therapeutic way, but I did not know how to translate that into action or what that might look like on the ground. After that, probably what happened was the pushing away rather than the welcoming of it, because there was a sort of sense of I do not know where this leads if I dwell too much on it or if I investigate it too deeply and probably also some fear of if I really get activated by this stuff, then my life might change and other parts of me not being sure if I was ready for that to happen yet. Then coming into contact with XR, listening to those stories of real people and then after that heading for Extinction talk, allowing myself to access the grief more and more deeply. From that point on, my Buddhist practice, and I guess my therapeutic practice, being a really helpful container and allowing me to feel that without being overwhelmed by it, often very intensely and acutely, but not tipping over into frozenness or despair. Partly because my Buddhist practice had matured and partly because there was an avenue of action that was possible that I did not know about before (P4).*

These comments touch upon several important issues in relation to climate anxiety. First, the participant is able to critically discuss the relations between their more general anxiety (and depression) and their more specific climate anxiety (and climate depression) (for research on the topic, see, e.g., Mulligan et al., 2023). They show awareness that these can interact with each other. The participant describes a process where becoming environmentally active via Buddhism is also linked with developing skills to differentiate between various kinds of anxiety and cope with them. At the same time, the participant works through issues in therapy. This shows how religion, in this case Buddhism, can have a role alongside psychotherapy in the general management of anxiety and depression (*cf.*, Goldin and Gross, 2010), but it also shows how multifaceted the process of engaging with climate anxiety (and grief) can be. The participant was motivated by others to approach these difficult feelings and the life changes that admitting them and the climate crisis can bring. The participant themselves uses the word container: in addition to Buddhist practice and therapy, it seems that the activist group also functioned as a container. The participant testifies that it is possible to live with these emotions instead of trying to repress them (“welcoming it,” “allowing me to feel that without being overwhelmed by it”), but many methods were needed for this. Merely activism or just therapy would not have been enough for them, but instead what they considered adequate was a combination of Buddhism, activism, and therapy.

One participant connects climate anxiety particularly to local environmental destruction.

*The lived experience of climate anxiety and emergencies is mostly pronounced here in Australia in bushfire season. It’s very strong. On one occasion, you were by the beach, but even here in Melbourne, this is the 2019 bushfires […], the bushfires is the smoke coming to the city. This creates a big jolt in the body. We have to do something. This is serious in that way. That was very personal, it’s a lived experience. […] Of course, we have had two hours away significant Black Friday, Black Saturday bushfires where 200 people died. They got stranded in a massive bushfire there. Even more recently, we have got floods in Victoria here, which is about 200 kilometres away, 100 kilometres away, totally destroyed communities with floods. Does this create anxiety? Not so much anxiety that’s debilitating, but it creates awareness that mobilises (P12).*

This quote is closely related to the ongoing discussion about what kind of terms should be used of the various psychological impacts of environmental issues. The participant links climate anxiety with local and regional disasters, showing the interconnections between climate anxiety research and disaster response research (for literature on this, see Celermajer, 2021). It is noteworthy that the participant emphasizes the practical eco-anxiety which resulted. The theme of local impacts of global climate change leads directly to the next topic, climate grief.

Overall, our results thus show that climate anxiety was discussed by several of the respondents, but it was not a dominant theme. Instead, they mostly discussed a wider variety of climate emotions, although many of these are closely related to climate anxiety.

### Grief

Feelings of sadness and grief arise in response to felt losses. They can help to engage with the losses and help to learn to appreciate what remains, but the process can be difficult and requires various kinds of coping methods with grief (e.g., Worden, 2018). Grief and sadness in relation to environmental changes have been called with various terms. Kevorkian (2004) proposed “environmental grief.” Albrecht et al. (2007) created the term solastalgia to refer to place-based feelings of sadness and longing, and forms of solastalgia have been studied around the globe (Galway et al., 2019). Since 2018, the term “ecological grief” has been prominently used, with climate grief being a major subject of study in relation to it (e.g., Cunsolo and Ellis, 2018; Benham and Hoerst, 2024). Ecological grief can arise both in relation to local events and global environmental change (Pihkala, 2024a).

As mentioned above, there has been a strong Buddhist influence in raising awareness on ecological grief, especially via Joanna Macy, who captures grief and other difficult eco-emotions under the term “pain for the world” (e.g., Macy and Brown, 1998). As was hypothesized, the participants had much to say about ecological grief, and several of them recounted grief-related activities they had participated in, or even developed themselves.

Several participants report experiencing ecological grief, many considering it to have been overwhelming at times. One participant (P2) reports feeling intense pain when witnessing environmental destruction locally or globally, and for this to be even stronger than empathy toward pain in other human beings or animals. Interconnections between ecological grief and having a strong environmental identity (e.g., Clayton et al., 2021) became visible in many responses.

The participants mentioned many objects of ecological grief. The global ecological and climate crisis was naturally a major object, but four participants mentioned also other, more exact sources of sorrow. One participant felt grief because of humanity’s common disconnect from the more-than-human world (P9)^5^, while another grieved over humanity’s greed and interpreted this in light of Buddhist teachings about karma.

*I do feel grief. Sometimes I just start crying. […] Now we have got spring, we have got many beautiful blossoms and flowers. I can be very moved sometimes and think, I have a grandson, maybe he will not see this. I can have the grief of also the tragedy that we humans fucked up so badly in what we did. I see it motivated mainly by greed. The Buddha taught us you have to renounce. But the Buddha understood that in samsara there’s greed and it’s never satisfied. The situation that we created is simply samsara. If it leads to the extinction of the human race or all the species on the planet, nature will not die, nature will still be here, but we will not. That’ll be the karmic result of everything that we humans in general have done. I have a lot of grief about that, and […] the emotional content of my Dharma practice is very involved with that (P1).*

In line with this quote, many participants (e.g., P1, P2, P3, P9, P13) testify to the difficulty of ecological grief, because the losses are so intense. They engage in various ways with Buddhism in order to cope with ecological grief. One participant links the severity of ecological grief with the anxiety-producing realization that political leaders could do major things for the environment, but they do not (for climate anxiety by youth because of this, see Hickman et al., 2021). The participant was horrified by the 2011 Mexican Gulf oil spill and hoped that President Barack Obama would respond by more progressive climate politics.

*For me, this was an incredible moment where you had birds dripping with oil, I mean, just like this horror, and he could make something of it, and he did not. I was heartbroken. I was really devastated. Heading classic Buddhist narrative I really started to lean into my practice rather than fall into despair. This was a way that the practice for me individually, just right here […], to work with that grief, and to not become bitter, to see that this was a moment with a long history that causes and conditions. […] It wasn’t about this one person, Obama, or this one legislator, Republican obstructions or anything like that. I noticed just how helpful it was to me (P10).*

Thus, the participant testifies that Buddhist practice was essential for their coping with ecological grief. Overall, many participants (e.g., P1, P2, P3, P4, P9) describe using Buddhist meditative practice to recognize and accept ecological grief, cultivating a sense of equanimity around it. One participant describes a very progressive method of engaging with ecological grief: developing a Buddhist inspired grief ritual. In the ritual they perform each morning, they first commune with the trees in their garden, and then ritually place stones on the table in their garden shrine representing the 200 species lost to extinction each day. The stones form a spiral, and once they reach the end of the table, they will start the spiral anew, replacing each stone with a larger stone. The practice is accompanied by sitting meditation and a prayer on behalf of the lost species (P2).

The participants described highly interesting dynamics in relation to the difficulty of starting to be in touch with ecological grief, illustrated by the following two examples. First, a doctor and researcher reflected on how emotion norms and common methods of coping via avoidance affected them:

*For a long time, I did not reflect on what it really means to go extinct. That was something that I only got more in touch with as I started doing the activism with DANCE and then Extinction Rebellion. I definitely remember, and that’s probably back to the whole Brexit thing […], 2019 when there was the general election and Boris Johnson got elected, the sense of hopelessness, that we cannot make a difference, there is this mega big problem but people are busy with their small nation state type issues. I have been experiencing those sorts of emotions, but for a long time, I’ve […] not been in touch so much with the grief of the loss of what’s at stake. […] Because I spent a lot of my professional life as a doctor and a researcher, it’s really easy to rationalize things and not have to feel the emotion, but just go about the evidence and the studies… (P4).*

Second, one participant testifies to becoming activated in response to witnessing the expression of ecological grief by an activist in an Extinction talk organized by Extinction Rebellion, realizing only at this moment how strongly they also felt environmental grief.

*Somebody who’d been coming to Extinction Rebellion for a number of months cried. She broke down and cried. For me that was the beginning of my career as an activist because I realized that I also had feelings. I was shocked to see her feelings. It was a surprise, and then something inside me recognized that emotion, and so I went on to experience the first, my first climate grief, I guess. It was the climate grief or love for the planet, both, or anger or frustration, that’s what fuels my action. If it wasn’t for that fit, connecting with that feeling, I do not think I’d be here, I do not think I’d be here at all. In the XR talk, they said we welcome your grief. That was also important, there was that explicit invitation or acknowledgement that people did have those feelings that was quite new for me. Then I did go to the next Rebellion (P5).*

The participant found a way to practice activism which felt suitable for her in XR Buddhists. They say that it has helped with difficult climate emotions, but at the same time it takes a lot of energy, bringing up the need to seek balance (*cf.*, Pihkala, 2022b).

Overall, our results thus show that ecological grief was a prominent topic for many of the respondents. Ecological grief had many objects and it was experienced as difficult, but Buddhism helped to cope with it. The influential Buddhist-inspired methods of encountering it (e.g., Joanna Macy) evidently had a role in this.

### Anger

Grief and anger have profound connections. In various kinds of grief processes, there often emerges feelings of anger because of the loss. For religious persons, these may become directed toward objects of belief, such as anger toward God in monotheistic religions (Exline et al., 2012; Pargament et al., 2000). Anger and blame are connected with interpretations of culpability and responsibility: people blame either others or themselves for causing the loss (e.g., Worden, 2018; Li et al., 2015). In relation to ecological issues, these interconnections between ecological grief and anger are profound, because ecological losses are caused by actions of humans, and the force of the losses can evoke feelings of anger toward objects of belief (Pihkala, 2024b). Indeed, among our participants, the same social and ecological issues could cause both grief and anger, but anger was much more complicated for them to engage with, as seen below.

For a long time, anger about ecological issues was a seriously under-researched theme. During recent years, there has been a growing interest toward especially climate anger (Antadze, 2020; Bergman, 2023), but empirical studies are still relatively scarce (for a review until 2022, see Pihkala, 2022a). In an innovative recent research article, Gregersen et al. (2023) studied various forms and objects of climate anger among Norwegians. Their results confirm that social and cultural factors shape interpretations of climate anger in profound ways (see also Du Bray et al., 2018). Religion is one of these factors, and philosophers of anger have pointed out that many religions, including Christianity and Buddhism, have usually profiled anger as something negative, leaving a confused legacy of downplaying constructive moral outrage and sometimes endorsing anger and violence in the form of religiously inspired wars (e.g., Srinivasan, 2017; for a discussion of anger, moral psychology and Buddhism, see McRae, 2018).

To give a summarizing introduction to the role of climate anger among our participants, virtually all of them mention anger in relation to Buddhism and climate activism, either experiencing various forms of it themselves or observing it in other Buddhists or activists. Anger is a major point of negotiation among the participants, and they represent a very diverse set of positions and interpretations on the theme.

Many participants recount earlier negative experiences of anger in activism in non-Buddhist contexts (e.g., P2 criticizes “shouty” or “accusatory” anger; P4 criticizes prior protests for being “fuelled by what felt like a lot of anger”). This made some participants (P2, P4) want to avoid activism altogether. They then describe having received transformative experiences of climate activism with other Buddhists, with other faith groups, or in Extinction Rebellion, because these featured no blaming as a principle and activist regenerative practices (regenerative culture), lacking this aspect of hostility they considered difficult.

Related to this aspect, many participants consider activists to often be too externally focused (e.g., planning actions) and that inner work, cultivating and coming from a positive mental state, should be emphasized more (e.g., P1, P3, P4, P9). Some describe Buddhist sitting meditation practice during actions to be particularly useful for serving as a container for difficult emotions without reactive outbursts of anger, as well as introducing a sense of stability and equanimity to the entire protest atmosphere. Many describe other activists and onlookers spontaneously joining them or reacting to them positively.

*In practice on the ground, in Rebellions, people seem to really like our Buddhists. Quite a few times, we have done walking meditation through the chaotic, different places where Rebellions [are] happening and people bow at us or give us a thumbs up, because we bring something quite different. There’s a lot of noise at Rebellions […] and the Buddhists counterbalance that quite nicely. There’s something about that sitting still and solid that I think is really important, and other people have said that too. (P5).*

Some of the participants have acted as teachers or counselors facilitating sharing, discussion and meditation events to resource other activists or help them debrief, and many consider Buddhism to offer have valuable tools to offer activists in this regard (e.g., P1, P3, P10). Nevertheless, many participants (e.g., P3, P5, P9, P10) highlight that they want to avoid Buddhist exceptionalism and consider such approaches and practices to also be found within the broader Extinction Rebellion framework and in other wisdom traditions.

The expression of anger in Buddhist communities is a topic of negotiation for the participants. Many consider Buddhists to avoid difficult emotions, difficult topics, and confrontation (e.g., P2, P9, P10). One of the reasons is that anger is considered to be one of the three mental poisons giving rise to suffering in human beings and elsewhere (samsara). In this context, suffering originates from an incorrect view of reality as composed of separate, permanent entities and selves, as well as attachment coupled with this misunderstanding (i.e., wanting things to be or not be a certain way and becoming angry when this is not the case). Another reason is a potential valuation of harmony and humility in Asian cultures where Buddhism originates (e.g., Gelfand and Denison, 2020), generating a norm of non-confrontational behavior and repression of negative emotions in Buddhist communities (see also McRae, 2018). However, it bears noting that such notions should be examined critically as they can also represent problematic cultural stereotypes and the legacy of Orientalism, and not be generalizable across diverse Asian Buddhist cultures. Western Buddhism is also described as often focusing on self-betterment through a study and meditation focus, and traditional Buddhism as focusing on self-transcendence and otherworldly goals (good karma, good rebirth, enlightenment) through merit making and spiritual practice. Both approaches can make the Buddhist practitioner orient away from the external world and toward the self and inner work, with external conflict considered to be a disturbance to such inner-focused practice. Furthermore, one participant (P9) identifies Western patriarchy and white, middle-class privilege as having a history of repressing expression of anger that arise in oppressed groups or that feature in the typical cultural expression in lower classes or other ethnic groups, and that this feature can also be seen in the culture of Western Buddhist communities. This aligns with the analysis by thinkers such as Srinivasan (2017) and Cherry (2021).

In general, most participants consider avoiding anger and confrontation in Buddhist belief and communities to be an obstacle for social, including climate engagement. However, the participants represent a wide array of viewpoints on the issue. One participant (P3) describes anger as a dangerous emotion that can easily lead to reactive and improper behavior even when the person considers to be acting out of anger in a beneficial way, and for self-restraint to be necessary to prevent the expression of anger. One participant (P1) describes sympathizing with anger in face of the ecocrisis, and even limited violence to property in other activists, as well as having felt anger themselves. They had reflected on the Extinction Rebellion rally cry “love and rage,” leading to a re-evaluation of the role of anger in activism, considering it to potentially also have a more positive function.

*Anger in relation to fossil fuel companies or banks that support fossil fuel extraction, or governments that support the system that allows this to continue, for me, anger against that is completely understandable. […] I do not reject people who get angry about it. In Extinction Rebellion, they often use the terms love and rage. Rage has a slightly different meaning from anger, but it’s still basically anger. I always thought that love and rage are two contradictory forces. But Extinction Rebellion seems to want to embody both in one approach. I quite like that in a way, and I think there is room for that. (P1).*

Many participants use the term “fierce compassion” to describe compassion motivated rage or moral outrage, or channeling anger into productive action through compassion (e.g., P1, P2, P3). Some described this anger as not being passive or targeted against individuals but rather an active energy to remove behaviors or bring down social systems causing socio-ecological harm. The participants connect such fierce compassion with either the Mahāyāna Buddhist compassionate practitioner ideal of the Bodhisattva who uses skillful means sometimes appearing wrathful on the outside, or to Tibetan Buddhist tantric practice of transforming negative mental forces into positive ones (on fierce compassion, see, e.g., Quaglia, 2022). An extreme case is illustrated by one participant (P7; they/them) who refers to Jātaka tales describing self-sacrificial virtuous behavior by the Buddha in his past lives as Bodhisattva involving causing harm and even killing other beings. They describe that the Bodhisattva committed these deeds out of compassion to save other beings, and was therefore willing to carry the negative karmic consequences of these acts. In this manner, the participant derives a justification for radical climate activism, including use of violence, from Buddhist teachings and examples on compassion. They also refer to more contemporary examples of sacrificial self-immolation out of compassion by Tibetan (Free Tibet movement) and Vietnamese Buddhists (peace movement during Vietnam War). One participant describes the potential for the use of fierce compassion but ends discussion on the theme by emphasizing the precedence of more gentle forms of compassion toward those suffering in face of the ecocrisis and socio-ecological collapse (P1).

Some participants describe being inspired and impressed by the example of moral outrage by public characters such as Greta Thunberg, and for this to have increased climate awareness also globally (e.g., P9, P11). Some describe confrontation to be essential for system change and therefore to be the truest manifestation of compassion. In this context, Buddhists deferring to gentleness are even described as using gentleness as a spiritual bypass, lacking compassion and wisdom, and representing a self-serving and comfort-seeking approach to Buddhist practice. Among two participants who engage in other forms of climate engagement rather than climate protests, one (P10) considers the confrontational side of the climate protest movement to be simply absorbed by the social system without effect, having developed an “immune response” to non-violent direct action through a succession of historical protests. Another non-protesting participant (P12) considers confrontational aspects of climate protests to create an adverse counter-reaction in the public and to be counter-productive to the cause.

The participants mention many subjects of climate anger, and they also describe anger in relation to their personality, family history, and social context. One participant (P3) describes coming from an emotionally repressive and non-confrontational family background, making it difficult for them to express anger. One participant (P4) describes anger about insufficient climate policies and discounting of scientific facts regarding climate change to be socially most suited to their background and social context as a medical doctor. Because of this, anger represented their primary emotional response to the climate crisis and being in denial of other climate emotions until deeper Buddhist practice and climate activism. One participant (P5) describes being confrontational by nature and has little internal struggles with experiencing and expressing anger. They also describe similar people to be enriched in activism, as well as being personally inspired by very provocative actions by other climate activists leading to arrest, jail sentences, and disputes among activists. Therefore, they seem to align with the confrontational Western activist tradition, and in agreement with this, they describe difficulties in relating to the meditative focus and nonconfrontational approach of other Buddhists and even other Buddhist climate activists. Their Buddhist tradition and practice is also less inwardly focused and virtue ethics based than the Buddhist practice of most participants.

Overall, anger thus emerged as a complex topic of negotiation for these Buddhist respondents, and we will return to this in the conclusions.

### Care and connection

Most participants mention compassion as a key motive for climate engagement.^6^ Compassion tends to be tied to interconnectedness in Mahāyāna Buddhism, and most participants also connect the two, sometimes referred to as compassion and wisdom representing the two reciprocal dimensions of Buddhist practice. The participants also often describe experiences of nature connection in terms of interconnectedness. Interconnectedness describes the Buddhist doctrine that all conditioned phenomena, including not only external and physical phenomena but also psychological (perceptual, cognitive, and emotional) phenomena, are intricately interconnected, co-arising and co-existing.^7^ Buddhist ecophilosopher Joanna Macy has likened the Buddhist understanding on interdependence to a scientific understanding of ecology and systems thinking (e.g., Macy, 2009). The existence of interconnectedness facilitates a sense of ethical responsibility, highlighting how human wellbeing is intimately tied to the wellbeing of other humans, other animals, and the biosphere. However, compassion toward the suffering of other living beings is considered to be needed to enhance the sense of ethical responsibility facilitated by understanding interconnectedness. Macy advocates extending such compassion to cover the entire biosphere, as the lives and wellbeing of countless living beings depend on a well-functioning biosphere. Macy has coined the term Ecosattva to represent an Eco-Bodhisattva, an environmentally aware Buddhist practitioner who seeks to practice Buddhism in order to save ecosystems maintaining all life.

Many of the study participants (P1, P2, P9, P10, and P12) mention Macy and combine compassion and interconnectedness in the manner advocated by her and other notable public Buddhist figures such as the Dalai Lama and Thích Nhất Hạnh. Most participants attribute lack of compassion (emotional disengagement, intellectual Buddhism) or a deficient understanding of interconnectedness as primary reasons for climate disengagement among Buddhists. One participant mentions that even those Buddhists who are concerned solely with directing their compassion toward beings becoming liberated from samsara rather than trying to improve it should take care of ecosystems, as healthy ecosystems are a prerequisite for Dharma (Buddhist teachings) practice (P3: “there is no enlightenment on a dead planet”).

There is considerable variability among participants in the particular understanding of compassion and interconnectedness and their relationship. Some participants describe extending compassion particularly to other human beings in the context of the socio-ecological crisis, considering the suffering of those in more affected regions (e.g., malnourished children; P11), or the suffering of also future human generations (e.g., P4). Others also extend the compassion to cover the current or future suffering of other animals or living beings in general, which are sustained by ecosystems undergoing destruction and collapse. This connects compassion with anticipatory ecological grief (for analysis of it, see Pihkala, 2024a). Five participants connect compassion to an animistic worldview inspired by the Buddha Nature doctrine in Mahāyāna Buddhism, formulated by medieval Japanese Zen master Dogen as also encompassing inanimate objects such as rocks and rivers, or indigenous worldviews. In this context, respect, care and compassion toward the non-human world arises from a sense of aliveness in all of interconnected nature, with similarities to Creation Spirituality and the Gaia hypothesis of the biosphere as one self-regulating system (*cf.* Mother Earth) (see, e.g., McFarland-Taylor, 2005).^8^

The modes of compassion described and advocated by participants vary from gentle and caring modes to fierce, subversive and radical modes. In the most extreme case, even violent and lethal action and bringing down destructive social and economic systems are justified in the name of compassion and interconnectedness, although most participants are highly critical and wary of violence and aggression in activism (see also section Anger).

Owing to the centrality of compassion in Buddhism for virtually all participants, interpretations on compassion are linked to many other Buddhist doctrines besides interconnection, to views on appropriate forms of activism, and to the other prominent environmental emotions. From the perspective of the doctrine of karma, actions performed from a mental state of compassion are considered to result in positive karma, representing a compassion focused Buddhist approach combining virtue and consequentialist ethics. This means that the mindset of activism is considered particularly important in determining the effects of activism. This likely explains why many participants (e.g., P1, P3, P4) consider it to be important to perform activism in the spirit of compassion and equanimity. In contrast, activism performed in the state of anger is considered by some (e.g., P1) to represent a defiled type of activism that cannot lead to positive outcomes owing to the mechanics of karma. Intriguingly, since it is the mindset and not the action that determines the karma, situational and contextual compassionate action, or in Buddhist terms, applying skillful means (s. *upāya-kuśala*), can even lead to action that seems destructive and radical on the outside. Therefore, compassion and nonharming (first ethical precept in Buddhism) can be used to justify both avoiding confrontation and engaging in radical action depending on the target and interpretation of compassion, as well as the specific context of the action, mirrored by the diversity of views on this topic by participants.

In terms of its relationship to other major emotions, compassion is both contrasted with anger, representing a counterforce to it and correct intention in its stead (e.g., P3, P4, P7), and described as a force that can express itself in an externally wrathful manner or be used to channel anger into productive behavior (e.g., P1, P2). Regarding grief, compassion and care for the environment or the suffering in living beings arising from the socio-ecological crisis are described by some participants to give rise to and be intimately connected to grief (e.g., P2, P13).

Deep connections between caring and difficult eco-emotions have been observed by scholars. For example, Hickman et al. (2021) has pointed out that eco-anxiety is fundamentally “eco-empathy.” Fascinatingly, these participants are manifesting a Buddhist interpretation of care and compassion as foundations for various eco-emotions and as fundamental motivations for caring about the Earth. This resonates also with the views of scholars of religion, ecology, and psychology such as LaMothe (2022), who argue that care would be a better fundamental foundation for environmental work than hope, which is more contingent.

Overall, our results thus show that compassion is a multi-faceted and highly important issue for these respondents. Many name it as a key motivation for PEB, and they connect it with various other emotions and aspects, such as interconnection.

### Guilt

While the participants shared the common understanding of compassion as hugely important for environmental matters, they had much reservations about eco-guilt. Broadly, ecological guilt refers to feelings around environmental responsibility and culpability (Jensen, 2019). Sometimes the formulation ‘environmental guilt’ is used, and differentiations are made between it and environmental shame (Fredericks, 2021). Pihkala (2022a) further differentiates feelings of inadequacy, which are common in relation to climate issues (e.g., Hyry, 2019) but not exactly guilt or shame, and feelings of regret and remorse. Empirical studies on eco-guilt have built on various operationalizations of it, and there is an ongoing discussion in interdisciplinary research about related emotions and their various potentials for PEB (e.g., Aaltola, 2021; Nielsen et al., 2024).

Ágoston et al. (2022) have proposed a measure of eco-guilt and given names to several kinds of eco-guilt, such as “system maintenance guilt” which arises out of knowing that one is part of structures which damage the environment. Indeed, a central issue in discussions about eco-guilt has been the relationship between individuals and larger systems or structures. Various environmental thinkers have differing views about the emphases that would be needed in order to evoke and/or channel eco-guilt into productive outcomes. Some argue that individual actions should not be a focus, but instead structural change, while others argue that both are needed for social change (see, e.g., Aaltola, 2021; Bryan, 2021). These negotiations between individual and structural responsibility featured heavily among our participants, as well as issues around privilege.

The personal transformation of participants included a large array of lifestyle changes, with most preferring vegan or vegetarian food (although many had already done so before for animal welfare reasons), avoiding flying, restricting or avoiding driving, avoiding unnecessary consumption, using renewable energy, practicing various forms of reuse and recycling, and considering environmental affairs important in political voting behavior. Many had become interested in the Deep Adaptation movement (Bendell and Read, 2021), assuming a belief in the unavoidability of socio-ecological collapse, and had reflected on how Buddhism and other beliefs and practices could serve the purpose of humanity adapting to the collapse and complete restructuring of social and economic systems (e.g., P1, P2, P11). Some had developed tensions with family members who were less concerned or engaged with the ecocrisis (e.g., P5). The lifestyle choices had also become a major source of tension and negotiation for the persons themselves, associated with feelings of guilt and various rationales for justifying consumptive habits and compromises.

*What am I going to do with my working hours? I have such privilege to […], because of the skills that I have and the support that I’ve enjoyed in the networks that I’m part of, be able to do something like One Earth Sangha. But also, how am I going to relate to the consumptive culture? (P10).*

One participant differentiates themself strongly from individual eco-guilt and uses interpretations of Buddhism in efforts to justify this:

*I do not feel the guilt in the sense that some people do. For instance, I have not got an electric car. I’ve got a car that uses fossil fuel. If I have to travel, it might be the best way to travel because of the cost of train. This is just one example. If I drive somewhere, I do not feel guilty about it. Some people do. I understand that. I do not have room for guilt. Buddha never taught that you should feel guilt. It does not help. Guilt is just a form of anger against yourself. I do not find it helpful. Regretting what we humans have done, and feeling responsible to do something about that, that is a more Buddhist emotion. (P1).*

Manifesting a directly opposite view, another participant adheres to feeling eco-guilt because of their vehicle emissions:

*Speaking of guilt, being in the wilderness is important to me. The way to do that is to have a four-wheel drive, which is a big vehicle. The way to cross rivers with a big vehicle is to have a diesel vehicle. So I just recently bought a new four-wheel drive, which was the most fuel efficient one I could find, but it’s still, diesel four-wheel drive. So, I’m spewing guilt around, leaving a trail of guilt behind me with that. (P11).*

Several (P9, P10, P13) participants engaged in critical discussions about perceived inadequacies of Buddhism in relation to eco-guilt and one’s own privilege.

*I think Buddhism is overly simplistic in some of these regards, and we can learn from other cultures. There are better ways of relating to the unavoidable violence that is entailed in living and breathing and eating. — How do we give back as we necessarily take, is something that’s not part of our Buddhist cultures and ways of looking because we value blamelessness. Oh, no, how am I going to get to blamelessness when it comes to the Earth? I cannot. (P10)*.^9^

*I grapple with the impact that I actually have. If you looked at someone like me, and my consumption, the planet would be unlivable if people live like I do. I guess that this is a real dilemma at the moment. I think one of the reasons why I’m so busy in doing things outside of and in my community is partly guilt-driven for the privileges that I’ve had. (P13).*

Some participants were able to balance their individual feelings of eco-guilt by seeing them in relation to structural issues:

*There’s definitely the motivation of wanting to make reparation, acknowledging that I have also been complicit in lots of things that have harmed the planet. I do not blame myself so much. I went through a phase of feeling really bad about buying plastic or just feeling very guilty. I do not feel that so much now, because I know it’s a systemic problem. (P5).*

Overall, Buddhist views and practices on the virtue of simplicity and how desire in the human mind is a root cause of overconsumption were considered to facilitate sustainability transitions at both individual and social levels. Buddhist elements featured less in justifications for consumptive habits and compromises, which participants occasionally acknowledged were based on personal desires and which were associated with guilt. One Buddhist inspired justification for unsustainable practices was referring to the ecocrisis as resulting from collective karma and its mitigation requiring collective responsibility, akin to the Western environmentalist theme of referring to the climate crisis as a systemic and not individual problem (P1, P5, P7, P8, P12).^10^ Considering individuals as only small contributors allowed some participants to justify moderation in personal lifestyle choices rather than going to lifestyle minimalist extremes (e.g., P1, P2, P10, P13), although some still felt conflicted about the compromises they were making (e.g., P2, P13).

### Despair, hope, and other future-related emotions

Most report feelings of despair, hopelessness, disappointment, or paralysis regarding climate activism and the ecocrisis (for these eco-emotions in general, see, e.g., Albrecht, 2011; Pihkala, 2022a; Greenspan, 2004). They use Buddhist views and practices similar to grief (e.g., view on impermanence, practice of meditation, equanimity, and letting go of grasping), to contain these emotional states and regain a sense of mental balance. They also use psychosocial support from other Buddhists and climate activists to in expressing these emotions and feeling understood and not being alone with their experience.

The participants relate these difficult feelings, among others, to perceived deficiency of local or global political and corporate sustainability policies and lack of efficacy of their climate activism and lifestyle choices. Some participants feel so uncertain about how to best affect change that this, when coupled with a strong desire to affect change, has resulted in strong feelings of paralysis and confusion (e.g., P3, P9). Many of the participants (e.g., P1, P2, P5, P6) have accepted that large-scale socio-ecological collapse is inevitable, some becoming acquainted and adopting a Deep Adaptation approach beginning to focus more on ways to adapt to the ecocrisis rather than ways to prevent or mitigate it.

Some participants (e.g., P1, P2, P5, P13) cope with the despair by focusing on what they can personally do, however little that may be, reclaiming a sense of agency in that. This resembles previous discussions of active and radical hope in climate activism (e.g., Active Hope by Joanna Macy and paper on active/radical hope among XR climate activists; see Macy and Johnstone, 2012). There are also participants who believe more strongly in the efficacy of climate activism, being inspired by success stories in the history of Western activist movements (*cf.*, Johnson and Wilkinson, 2020). Several participants (e.g., P1, P2, P5, P10) consider a sustainability transition by humanity to require a transformation of consciousness to remove the desire driving overconsumption and exploitation of resources, and for Buddhist views and practices to offer a suitable foundation for such a transformation. They find hope and agency in participating in and generating such root level transformation even if little externally evident or rapid progress occurs in socio-ecological systems (for similar views and discussion, see, e.g., Pihkala, 2018; Wiseman, 2021). This also allows the participants to feel empowered by and contented with focusing on Buddhist practice despite the ongoing ecocrisis, potentially representing one way to justify inaction and protect the self from feelings of guilt and disappointment. Intriguingly, despite considering Buddhist practice to produce transformation at the root, most participants consider Buddhists should get off the meditation cushion and engage more strongly with the climate crisis (*cf.* Javanaud, 2020). The observed inactivity of most Buddhists combined with the green transition potential seen in Buddhism causes many of the participants to have a mixed bag of views regarding the ability of Buddhists to affect change. One participant considers Buddhism to be good at resourcing activists (pastoral aspect) but poor at speaking out about the difficult truth of the climate crisis (prophetic aspect) as well as at organizing communities for change (practical aspect), such as the green transition or climate activism.

Interestingly, many participants do not consider hope to be a prerequisite for considering activism meaningful. Several participants (e.g., P2, P3, P10, P11) describe experiencing a strong sense of moral alignment when participating in climate activism, representing behavior that aligns with their values. Many participants (e.g., P2, P3, P8, P11) report strong feelings of nonseparation, congruence and being true to themselves during actions, sometimes involving sitting meditation. Two participants in their 70s (P1, P11) describe an intention to spend the rest of their lives acting out of such alignment, regardless of whether it leads to external change. Therefore, a sense of acting out of moral alignment seems to provide a powerful sense of meaning and agency, emotional relief, and strong positive experiences to participants in and of itself. Such experiences and sense of meaning allow activists to transcend hope entirely. Such transcendence of hope can be considered to fall within prior concepts of “active” (Macy and Johnstone, 2012), “radical” (Lear, 2006), or “wise” hope (Halifax, 2022), also reported among Extinction Rebellion activists in general (Stuart, 2020). Alternatively, it could be considered to feature the distinct quality of being wholly unconcerned with efficacy and the issue of hope in favor of a mental orientation focusing solely on positive intentions or experiences of moral alignment as a foundation for activism. This resonates with recent research on various motivations of environmental activists, some of whom practice “non-hope” (Cassegård, 2023; Malmqvist, 2024; Wettergren, 2024).

### Uplifting emotions

Many of the participants describe finding major psychosocial support particularly from other Buddhist climate activists, while describing difficulties in relating to non-Buddhist activists (e.g., too much aggression or external focus) and non-activist Buddhists (e.g., approach too self-centered or socially passive, or lack of awareness or concern over climate crisis). Other Buddhist climate activists were often from different Buddhist traditions, but despite this, many of the participants considered the overlap of values and approach to activism to unite them in a unique and highly meaningful manner. Most participants describe deriving a positive sense of togetherness from Buddhist or climate activist communities in isolation from each other as well, allowing communicating and sharing key values and experiences and engaging in meaningful activities as part of a group. Being asked to join a socially engaged group or activist event by a family member or friend, or being inspired by the example of activism by family members or friends, influenced the activation of many of the participants. Nevertheless, most participants also reported individual motives and climate awakening as motives rather than social factors alone. Nevertheless, some participants (e.g., P5) describe having experiences of joy and fun in activism (for various uplifting climate emotions, see Pihkala, 2022a; Ray, 2020)^11^. One participant (P5) considers disengaged people to often see climate activism as emotionally and physically difficult, contributing to their unwillingness to engage. She considers this unfortunately since in her experience there is also a lot of fun and joy involved in designing and performing actions. However, fun and joy were not interpreted by participants in light of Buddhist views and practice. It is possible that they are considered to be innocent pleasures rather than either spiritual merits or demerits warranting negotiation.

## Conclusion

This study has analyzed the complex negotiations around climate emotions among 13 Western Buddhist climate activists. As seen above, while the number of study participants is small, the responses of these individuals in special positions reveal highly interesting dynamics of climate emotions and the ways in which Buddhism is negotiated in relation to them.^12^ The results contribute to research knowledge about climate activism, emotions, and religion.

The study participants verbalized a wide range of emotions (using a broad definition of emotions including feelings) in connection with climate change. Many of these emotions are familiar from earlier research and support the notion that particular emotional landscapes are central to the human response to climate change, as well as among climate activists in particular (e.g., Poma and Gravante, 2024). Nevertheless, the specific ways in which the participants elaborated on these emotions reflected their educational and cultural as well as their Buddhist background (see also Sauerborn, 2022). One particularly striking example is the wide-ranging use of the concept of compassion, central to Mahāyāna Buddhism and Buddhism in general, in connection with numerous climate emotions, including climate anger, grief, connection, and care. Conceptions of compassion permeated the expression and interpretation of climate emotions by the participants, and compassion emerged as a key climate change related emotion in itself.

As was expected, many of the study participants had certain special skills in relation to some climate emotions. Especially encounters with climate grief and despair benefited from Buddhist-inspired methods, such as Joanna Macy’s Work That Reconnects, and some of the participants had developed methods with which they helped others in relation to these emotions. Grief had also inspired some to become more active, and thus our study confirms for its part the political potential of ecological grief (Cunsolo and Landman, 2017). However, many climate emotions were still challenging for many of them, and thus our study points to the need in climate emotions research to explore emotion-specific skills in addition to general coping and emotion regulation skills.

Climate emotions were connected with pro-environmental behavior (PEB) and environmental activism in profound but complex ways. Many participants (e.g., P2, P3, P4, P11) reported a eudaimonic concept of happiness (seeking to act in accordance with one’s values), linked to Buddhism (e.g., Ekman and Simon-Thomas, 2021), which played a key role in PEB and climate activation.^13^ Climate activism was motivated by seeking to live according to one’s values as well as to cope with climate emotions rather than by hope for change, supporting the importance of non-hope-related motives for climate activism as discussed recently also by others (e.g., Cassegård, 2023; Stuart, 2020). Nevertheless, activist burnout and feelings of despair (Bird et al., 2024) and eco-paralysis (see Albrecht, 2011) occasionally led to reorientation toward more traditional and less engaged Buddhist practice, with a focus on teaching and meditation. In general, our study confirms the view that many kinds of emotions, both those which are often labeled as “positive” and “negative,” can have constructive impacts on PEB (see also, e.g., Poma and Gravante, 2024; Sangervo et al., 2022).

The participants also struggled with anger in activism, which could lead to either turning away from activism or seeking to engage in activism in a mindset and in modes (e.g., meditation protests) characterized by equanimity and compassion. Denial of climate emotions and emotional blocks causing avoidance of the issue was also reported, although obviously the study participants (as climate activists) had (at least mostly) overcome these emotional blocks. Buddhist meditation practice played a role in helping acknowledge these emotions, but in the absence of climate activation, being aware of the emotions through Buddhist practice could exacerbate their perceived negative effect (as reported by P2 who found this effect of Buddhist practice prior to climate activation). Furthermore, complex questions remain around the impacts of these practices on constructive climate anger: does meditation put out its fire?

The data shows well that people may have various kinds of fundamental assumptions about the intensity and temporality of emotions. This can have significant impacts on how eco-emotions are understood. For example, the participants overall interpreted guilt to refer to rather strong emotional states, while scholars of eco-guilt often emphasize that eco-guilt can manifest also briefly and mildly (e.g., Jensen, 2019). The evaluations of whether a certain eco-emotion is productive in relation to PEB are affected by these kinds of fundamental assumptions about emotions, and indeed the participants had different views on the usefulness of climate guilt. Overall, it could be argued that many of the participants, and perhaps engaged Buddhism in general, would benefit from a wider understanding of various possible manifestations of (eco-)emotions and their contextual relationship with PEB.

These Western Buddhist climate activists manifest various levels of emotional granularity, the ability to name different emotions and emotional nuances (see, e.g., Barrett et al., 2001; Smidt and Suvak, 2015; Tan et al., 2022). Some participants had very high skills in this and differentiated, for example, between various forms of sadness-related emotions. Others operated with a more simple vocabulary, but in general the participants had clearly thought about various emotions also before, at least partly because of their Buddhist practices. Our results confirm the view in (climate) emotion research that increased emotional literacy and skills in emotional granularity lead into a wider array of options for coping and emotion regulation (e.g., Smidt and Suvak, 2015).

The data and our analysis of it point toward a larger issue in relation to emotional granularity and eco-emotions: there is clearly a need for a wider understanding of variations inside a general emotional term, such as grief, anger, or guilt. Ecological grief is not just about very strong mourning, but also about other kinds of sadness arising out of environmental changes. This is something which our Western Buddhist participants recognized quite well, but this seems to be a challenge more widely in societies (see the discussions in Cunsolo and Landman, 2017; Pihkala, 2024a). However, the participants had much more trouble in seeing variations of eco-anger and in recognizing the nuances of eco-guilt. This seems to point toward a major challenge also for a wider public and broader eco-emotion research: if people only think of anger as strong embodied anger and guilt as intense feelings of guilt, the interpretations of eco-anger and eco-guilt become restricted. For example, the constructive dimension in moral outrage about climate injustice may be left unnoticed and unpracticed, as was common for our participants. Moreover, the manifold manifestations of eco-guilt in everyday life issues about responsibility may be left unnoticed, too (Jensen, 2019), as well the important moral potential in guilt in general (Fredericks, 2021; Aaltola, 2021).

This study focusing on climate emotions fills important knowledge gaps regarding embodied Buddhist ecophilosophies and the relationship between Western Buddhist teachings and practice and pro-environmental behavior, as most of the literature on the theme to date has been based on doctrinal study alone. Based on the study findings, Buddhist interpretations of impermanence and solitary or communal meditative or ritual practices can be used to embrace difficult emotions related to destruction of the more-than-human world and to resource activism, preventing burnout. Compassion, on the other hand, can be used to engage with the climate crisis, counteracting human tendencies for indifference and compartmentalization. This may especially be the case when compassion as a value is connected to a eudaimonic concept of happiness where action is driven by seeking alignment with values, commonly reported among the study participants. Nevertheless, these doctrines and practices are typically harnessed to such purposes only in connection with a climate awakening brought about by non-Buddhist social contexts. The study participants report ‘non-climate-awakened’ Buddhists to also use Buddhist teachings and practices in opposing ways, justifying indifference about the climate crisis in the name of escape from samsara or focus on traditional modes of individual study and practice disconnected from societal concerns.

Guilt and anger emerge as somewhat conflicted themes. On the one hand, the Buddhist ethical precept of non-violence is critiqued for being incompatible with ecology and leading to unconstructive levels of individual guilt, and the concept of guilt itself is critiqued for being incompatible with Buddhist teachings. On the other hand, the concept of collective karma is considered to allow assuming a share of responsibility for humanity’s environmental behavior without crippling personal guilt. As one of the three root mental poisons (alongside desire and ignorance), anger is widely considered to be problematic, and Buddhists can have difficulties accepting and managing anger in themselves and among others, with mostly disincentivizing effects on participating in climate activism and discussing climate change, which is a difficult issue, in Buddhist communities. Some use traditional Buddhist teachings on fierce compassion to justify using anger for positive purposes, while others call for a more positive attitude to anger in Western Buddhist communities, associating avoidance of anger also to the historical legacy and oppressive conventions of white middle class convert Buddhism.

By revealing how Buddhist views and practices influence the relationship between climate emotions and pro-environmental behavior among Western Buddhists, the study also has broader implications for environmental activism research and religious studies. First, even though contemporary environmental activism is often not primarily religiously motivated, religion plays a major role in how activism is conducted and interpreted. Religious emotion norms concerning conflict and anger can have a particularly strong influence on activation. Second, nevertheless, owing to the diversity of teachings, interpretations, and practices in any major religious tradition, adherents can selectively filter those teachings and practices that justify their individual emotional and practical engagement with the climate crisis. Therefore, religious interpretations can be used to provide emotional support for the individual regardless of their environmental stance, and can promote both disengagement and pro-environmental behavior.

There are several constraints concerning the generalizability of the research findings arising from the methodology and limited scope of the sample. First, the qualitative methodology featuring a mix of unstructured and semi-structured interviews allowed us to understand individual participants at a high level at the expense of more systematic comparisons facilitated by a structured set of questions. Second, the sample size is small, including only thirteen (13) participants, and does therefore not allow quantitative conclusions on the prevalence of the observations among Western Buddhists. Third, the participants are from Anglo-American countries, are mostly highly educated (minimum of bachelor’s degree from University), are all above 30 years of age, and only one participant represents non-Caucasian ethnicity. Therefore, even among Western Buddhists, the sample is demographically biased, which likely influences the study findings. For instance, younger people may display different types and intensities of climate emotions compared to adults (e.g., Hickman, 2019; Hickman et al., 2021; Nairn, 2019), which could influence Buddhist interpretations and practices related to climate emotions. Moreover, even the participants in this study exhibit local emphases (e.g., old forest logging in Canada and indigenous claims to land in Australia), which are likely to influence climate emotions and their interpretation. Therefore, the observations would likely have displayed greater diversity with the inclusion of also other non-Anglo-American (e.g., EU) countries. Fourth, we only studied activists, and including also non-activists could have richer material concerning de-motivating factors leading to social passivity. In the current study, our material on this aspect was limited to the activists’ personal experiences (e.g., negative relationship with activism in the past) and views on disengaged Buddhists, which may be, for instance, negatively biased.

Fifth, the study omits Buddhists in Asian countries where approximately 99 % of Buddhists are estimated to reside (PRC, 2012). A key reason for this was the difficulty in identifying Asian-based Buddhist groups participating in climate protests through English-language websites and networks. Because of this, the study findings should be interpreted in the particular context of (modernized and contemporary) Western Buddhism, and cannot be generalized to Asian societies, where societal structures, cultural conventions and norms, environmental attitudes and movements, and Buddhist beliefs and practices, among other relevant factors, can all differ considerably from those in Western societies. Because of the study limitations, future research priorities include broadening the focus to cover both active and non-active Buddhists from diverse cultural settings and backgrounds, particularly from Asian societies and Asian immigrant communities. Future research should also explore linkages with research on climate emotions among activists from other religious and spiritual traditions (e.g., for reflections on Christianity and climate emotions, see Malcolm, 2020).

Overall, the examples provided by these active people give much to think about in relation to research on environmentalism, religion, and emotions. The data shows how many different emotions can be present in climate activism, and how religious interpretations may shape engagement with them. Amidst an intensifying climate crisis, attention to such dynamics is one important part of resilience-building.

## Data Availability

The datasets presented in this article are not readily available because the interview data contains sensitive information on political activism, including criminal activity. Moreover, much of the data is life story interview data which cannot be anonymized without removal of the bulk of the data owing to a high density of information such as events and social connections which may allow identifying the study participants. To protect the study participants, we have decided against sharing the data in a public repository (see e.g., Näre, 2022). Requests to access the datasets should be directed to johannes.cairns@helsinki.fi.

## Footnotes

^1^We use the term “eco-emotions” to refer to emotions which are significantly connected to environmental issues, and “climate emotions” to refer to those of them which are significantly connected with anthropogenic climate change. For discussions on terminology, see Pihkala (2022a, 2024b).

^2^By “world religions” we here refer to religions with a large following, such as Christianity, Islam, Hinduism, and Buddhism, rather than religions traditionally listed as world religions, a concept (world religion paradigm) that has attracted criticism due to roots in colonialism and various biases (see, e.g., Benson, 2020).

^3^The Yale Forum on Religion and Ecology, currently operating under the Yale Center for Environmental Justice, was founded in the United Nations in 1998. The project seeks to facilitate academic exploration on how religions may contribute to environmental solutions as well as religious environmentalism (see https://fore.yale.edu/, Accessed 2024-03-21).

^4^We did not study non-verbal manifestations of climate emotions, since that would require a different methodology. For that kind of research, see, e.g., Hufnagel (2018).

^5^“*For me, it’s grief and it’s sadness*. It’s thinking of the suffering that people are and will experience and the magnitude of it, and then the loss of species and other forms of life. Maybe also this *disconnection*, that we are so disconnected, and I’m still pretty nature disconnected. I try to be more nature connected. I know it’s good for my health when I can do it. I spent a lot of time inside in front of this little screen. But just sadness, sadness of why we cannot get it together. […] I call myself a Buddhist because that’s easy to define but […] I’m sort of eclectic spiritually beyond Buddhism too. […] But that’s also what I feel in a Buddhist context and when I really get down, it’s this *immense sadness*. […] Personally, the more emotion is this *longing for the nature connection*. The grief is more thinking of the bigger picture. It’s not about my own life.” (P9).

^6^Especially in Mahāyāna Buddhism, covering East Asian Buddhism, Tibetan Buddhism and most Western Buddhist movements, compassion is considered to be one of the main aspects of Buddhist practice. The ideal practitioner is a Bodhisattva who practices Buddhism with the compassionate intention to help all sentient beings become enlightenment, representing ultimate freedom from suffering in the cycle of samsara. Compassion (s./p. *karuna*) is also one of the four limitless mental states (*brahmavihara*) cultivated in both Mahāyāna and particularly in the other main form of Buddhism, Theravada Buddhism, alongside loving kindness (s. *maitri*, p*. metta*), sympathetic joy (s. *upeksha*, p. *upekkha*), and equanimity (s./p. *mudita*). However, loving kindness tends to be stressed more in the Theravada tradition relative to compassion, and (the modern Western form) of Theravada practice tends to be less focused on a developing a caring relationship towards other beings and more focused on personal liberation from samsara through enlightenment. Nevertheless, the centrality of compassion in most forms of Buddhism likely explains why practitioners use the word compassion to describe positive affects concerning ecosystems instead of words such as care and love. Similarly, experiences of nature connection are often described in terms of the Buddhist concept of interconnectedness.

^7^The doctrine of interconnectedness has some bearing upon early Buddhist teachings on the non-self, impermanent and composite nature of all phenomena. A later development occurring in India with the development of Mahāyāna Buddhism was the doctrine of emptiness (s. *shunyata*) formulated particularly by Nagarjuna, where all conditioned phenomena are considered to be totally void (i.e., empty) of any substantial (permanent, unchanging) basis, coarising, coexisting and coceasing. Approaching the teachings on nonself, impermanence and emptiness through the concept of interdependence was advocated particularly in the Huayan school of Buddhism originating in China. Classical texts in this school describe the interconnectedness of all phenomena through the Indra’s web metaphor where every jewel at the intersection of the strands of the web reflects all other jewels.

^8^Macy has co-operated with theologian Matthew Fox, a major leader in Creation Spirituality (see Kaza, 2020).

^9^Also: “The lived history of Buddhism is that it has always relied on wealth. […] Wealth has always entailed, or systematically, patternwise, entails ecological harm. […] It’s a convenient thing that we do in saying, Oh, as long as I did not kill the animal, I can eat the meat. - - I think reciprocity as a principle is much more viable, and entails action, entails necessary care, which is, of course, part of compassion, karuna. It’s in these teachings, but we need […] bring these in and make it more nuanced and complete. There’s an incompleteness to our analysis of ecological harm.” (P10).

^10^“Is there a guilt? That’s an interesting question. […] There’s a wonderful song here by an Australian rock band who have always been committed to social justice. […] There’s a line in the first couple of songs, something like, “We let you down, stop your toys, I want to talk to you, I want to tell you that we let you down and we are sorry. […] The beautiful thing about David Loy is that he talks about the personal dukkha, which is greed, anger and ignorance. He talks about how that can manifest in our institutions and in our structures. So it’s not just personal dukkha, it’s also structural dukkha. That’s a gift for us. Buddhism needs to pay more attention to the structural dukkha, which social work talks a lot about that. We need to see the structure and the process that’s influencing the personal suffering as well. […] We have to marry them together in a way that we feed each other because typically people in the social activist group, they do not have this piece [personal suffering]. They act from a dualistic perspective of conflict as opposed from oneness in that way.” (P12).

^11^A popular visualization, the Climate Emotions Wheel, mentions many of these uplifting emotions in a segment called “Positivity” (https://www.climatementalhealth.net/wheel).

^12^The climate emotions are connected to life paths and activist identities, which the authors focus on in a separate manuscript on the same data (Cairns et al., manuscript).

^13^Research on happiness can be classified into the hedonistic (seeking pleasure) and eudaimonic (seeking fulfillment, flourishing) traditions. In this scheme, hedonistic happiness is defined as the presence of positive and absence of negative affect, while eudaimonic happiness denotes a deeper satisfaction derived from living in a manner considered meaningful. Both concepts can be defined in numerous ways based on different philosophical and religious traditions and can overlap in various ways (e.g., Deci and Ryan, 2008).
